# Adverse birth outcomes and their clinical phenotypes in an urban Zambian cohort

**DOI:** 10.12688/gatesopenres.13046.2

**Published:** 2020-01-24

**Authors:** Joan T Price, Bellington Vwalika, Katelyn J Rittenhouse, Humphrey Mwape, Jennifer Winston, Bethany L Freeman, Ntazana Sindano, Elizabeth M Stringer, Margaret P Kasaro, Benjamin H Chi, Jeffrey SA Stringer

**Affiliations:** 1 Division of Global Women's Health, Department of Obstetrics and Gynecology, University of North Carolina, School of Medicine, Chapel Hill, NC, USA; 2 Department of Obstetrics and Gynaecology, University of Zambia School of Medicine, Lusaka, Zambia; 3 UNC Global Projects – Zambia, Lusaka, Zambia

**Keywords:** adverse birth outcomes, pregnancy, preterm birth, small for gestational age, stillbirth, sub-Saharan Africa, Zambia

## Abstract

**Background**: Few cohort studies of pregnancy in sub-Saharan Africa use rigorous gestational age dating and clinical phenotyping. As a result, incidence and risk factors of adverse birth outcomes are inadequately characterized.

**Methods**: The Zambian Preterm Birth Prevention Study (ZAPPS) is a prospective observational cohort established to investigate adverse birth outcomes at a referral hospital in urban Lusaka. This report describes ZAPPS phase I, enrolled August 2015 to September 2017. Women were followed through pregnancy and 42 days postpartum. At delivery, study staff assessed neonatal vital status, birthweight, and sex, and assigned a delivery phenotype. Primary outcomes were: (1) preterm birth (PTB; delivery <37 weeks), (2) small-for-gestational-age (SGA; <10 ^th^ percentile weight-for-age at birth), and (3) stillbirth (SB; delivery of an infant without signs of life).

**Results**: ZAPPS phase I enrolled 1450 women with median age 27 years (IQR 23–32). Most participants (68%) were multiparous, of whom 41% reported a prior PTB and 14% reported a prior stillbirth. Twins were present in 3% of pregnancies, 3% of women had short cervix (<25mm), 24% of women were HIV seropositive, and 5% were syphilis seropositive. Of 1216 (84%) retained at delivery, 15% were preterm, 18% small-for-gestational-age, and 4% stillborn. PTB risk was higher with prior PTB (aRR 1.88; 95%CI 1.32–2.68), short cervix (aRR 2.62; 95%CI 1.68–4.09), twins (aRR 5.22; 95%CI 3.67–7.43), and antenatal hypertension (aRR 2.04; 95%CI 1.43–2.91). SGA risk was higher with twins (aRR 2.75; 95%CI 1.81–4.18) and antenatal hypertension (aRR 1.62; 95%CI 1.16–2.26). SB risk was higher with short cervix (aRR 6.42; 95%CI 2.56–16.1).

**Conclusio** **ns**: This study confirms high rates of PTB, SGA, and SB among pregnant women in Lusaka, Zambia. Accurate gestational age dating and careful ascertainment of delivery data are critical to understanding the scope of adverse birth outcomes in low-resource settings.

## Introduction

The often overlapping outcomes of preterm birth (PTB), small for gestational age (SGA), and stillbirth (SB), collectively called ‘adverse birth outcomes’, are responsible for most perinatal morbidity and mortality worldwide. ^1, 2^ Low- and middle-income countries bear the overwhelming burden of global PTB, SB, and SGA. ^3– 5^ However, reliable classification and estimation of adverse birth outcomes in low-resources settings is challenging because of a number of interrelated factors, including (1) uncertain gestational age dating, ^6^ (2) conflation of fetal growth restriction and PTB into the less useful metric of ‘low birthweight’, ^3, 5^ (3) inconsistent thresholds for fetal viability, ^7^ and (4) misclassification of stillbirth and neonatal death. ^8, 9^ In many countries, including Zambia, data sources that adequately address these methodological challenges are lacking to the extent that national estimates of adverse birth outcomes must be modeled. ^3, 6, 10, 11^

In sub-Saharan Africa, cohort studies in pregnancy rarely use reliable gestational age dating or clinical phenotyping to classify outcomes. Deliberate clinical phenotyping that characterizes the events that incite parturition (i.e., spontaneous vs. provider-initiated), quantifies maternal and fetal co-morbid conditions, and reliably distinguishes the timing of perinatal death is essential for rigorous classification of adverse birth outcomes. ^12, 13^ Accurate estimation of gestational age with fetal ultrasound is also critical. Other dating methods, such as maternal recall of last menstrual period (LMP), ^14– 17^ symphisial-fundal height measurement, ^18^ or newborn physical exam ^19, 20^ introduce error (and in some cases, bias ^21^).

We established a cohort of 1450 pregnant women and their infants at a tertiary care institution in Lusaka, Zambia, with the goal of better understanding the epidemiological factors and biological mechanisms leading to adverse birth outcomes. This report presents the outcomes of the first phase of this cohort.

## Methods

The Zambian Preterm Birth Prevention Study (ZAPPS) is an ongoing prospective observational cohort study at the Women and Newborn Hospital of the University Teaching Hospitals (UTH-WNH), the primary referral hospital in Lusaka. Phase 1 of ZAPPS, the subject of this report, recruited and enrolled participants beginning in August 2015 and completed follow-up in June 2018. The sample size for this observational study was initially set at 2000 women, with a target of 250 preterm birth events based on published regional population estimates. ^4^ For budgetary reasons and because the prematurity rate was higher than initially expected, enrollment was stopped in September 2017 after 1450 women had been enrolled. The ZAPPS protocol was developed to align with the Guidelines for Strengthening The Reporting of Observational Studies in Epidemiology (STROBE). ^22^

### Study population

Pregnant women meeting the following criteria were eligible for enrollment in Phase 1 of the ZAPPS cohort: (1) 18 years of age or older; (2) viable intrauterine singleton or twin gestation; (3) presentation to antenatal care prior to 20 weeks of gestation if HIV-uninfected or 24 weeks if HIV-infected; (4) residing within Lusaka with no plans to relocate during the study follow-up period; (4) willing to provide written, informed consent; (5) willing to allow participation of their infant(s) in the study; (6) willing to be contacted and followed up at home if necessary.

The ZAPPS protocol was approved prior to study initiation and is subjected to annual review by the University of Zambia School of Medicine Biomedical Research Ethics Committee (reference number: 016-04-14) and the University of North Carolina School of Medicine Institutional Review Board (study number: 14-2113). The study also received approval from the Zambian Ministry of Health National Health Research Authority. Each participant provided written informed consent before enrollment.

### Procedures

Full study procedures are described in detail elsewhere. ^23^ Community educators identified potential participants at antenatal care clinics of UTH-WNH and five surrounding clinics in Lusaka, assessing basic eligibility criteria such as age and approximate gestational age. Interested volunteers underwent ultrasound examination per standard of care to determine pregnancy location, fetal viability, number of fetuses, and gestational age by standard biometry (Sonosite M-Turbo, Fuji Sonosite, Bothell, WA). Gestational age was calculated at enrollment by crown-rump length if <14 gestational weeks or by head circumference and femur length if ≥14 weeks. Fetal biometry structures were each measured twice and then averaged to calculate gestational age using INTERGROWTH-21st equations. ^24, 25^ Pregnancies below the lower threshold for INTERGROWTH-21st equations were dated by the Hadlock formula. ^26^ Interested women who met preliminary ultrasound eligibility criteria completed an informed consent process in their preferred language of English, Nyanja, or Bemba.

At enrollment, study nurses collected demographic and behavioral information through medical record review and participant interview, and documented a thorough health history including prior pregnancy outcomes. As part of standard antenatal care, participants underwent a physical exam and rapid testing for hemoglobin, urinalysis, syphilis (SD Bioline Syphilis 3.0, Abbott Diagnostics), and HIV (SD Bioline 3.0, Abbott Diagnostics).

After enrollment, participants received routine antenatal care at follow-up visits scheduled at approximately 24 weeks, 32 weeks, and 36 weeks. All participants underwent cervical length measurement in the second trimester (i.e., 14–28 weeks) and fetal growth assessment by biometry in the third trimester. ^27, 28^ Cervical length measurements were performed by sonographers with certification in the Cervical Length Education and Review (CLEAR) program. Study nurses staffed the UTH-WNH labor ward full-time and collected detailed information about the clinical course and perinatal outcomes of participants and their infants, including gestational age at birth, neonatal vital status, birthweight, and sex, and assigned a delivery phenotype. For participants who did not deliver at UTH-WNH or were not captured by study staff during their delivery admission, study staff collected perinatal outcomes either in person or by phone. Cohort retention in this analysis was defined as ascertainment of date of delivery.

### Exposures

Primary exposures evaluated included maternal age (years), height (cm), and body mass index (BMI, kg/m ^2^); reported prior preterm birth (nulliparous, parous with no prior PTB, or parous with one or more prior PTB); cervical length (mm) and short cervix (<25mm); gestation (single or twin); hypertension during pregnancy (≥140 systolic or ≥90mmHg diastolic at any antepartum study visit); anemia at enrollment (<10.5g/dL); bacteriuria during pregnancy (1+ leukocyte esterase and/or nitrites at any antepartum study visit); syphilis seropositivity (reactive at enrollment); and HIV seropositivity (reactive at enrollment).

### Outcomes

Primary outcomes of this analysis were: PTB, defined as birth between 16 ^0^/ _7_ and 36 ^6^/ _7_ gestational weeks, SGA (newborn weight-for-age <10th percentile by INTERGROWTH-21st norms), ^29^ and SB (delivery of an infant without signs of life ≥16 ^0^/ _7_ weeks). Secondary outcomes included very PTB (birth before 34 ^0^/ _7_ weeks), very SGA (newborn weight-for-age <3rd percentile), gestational duration (days), and birthweight centile for gestational age at delivery. Both PTB and very PTB were further characterized as either spontaneous (spontaneous labor or membrane rupture prior to labor) or provider-initiated (induction of labor or pre-labor cesarean). We differentiated antepartum stillbirth (i.e., fetal heart tones absent on admission or, if not assessed, maceration skin changes present at delivery) from intrapartum stillbirth (i.e., fetal heart tones present on admission or, if not assessed, absence of maceration skin changes at delivery).

### Statistical analysis

We performed descriptive analyses of baseline characteristics and exposures of the cohort, reporting median and interquartile range (IQR) for continuous variables, and frequency and percent for categorical variables. Differences in baseline characteristics between women retained at delivery and those lost to follow-up were evaluated by univariate tests of association.

We summarized parturition phenotype among our retained participants by preterm versus term and spontaneous versus provider-initiated following a standard rubric. ^12^ Among spontaneous PTB, we identified primary maternal, fetal, and/or placental conditions present at the time of delivery. Among provider-initiated PTB, we reported the primary indication for delivery as recorded by the provider. Finally, key individual conditions present and phenotypic clusters were used to classify all PTB, spontaneous PTB, and provider-initiated PTB.

We calculated the incidence of adverse birth outcomes: PTB, spontaneous PTB, very PTB, spontaneous very PTB, SGA, very SGA, and SB among all participants retained at delivery. Twin deliveries in which at least one neonate was SGA or stillborn were classified as having met the respective outcome. Crude associations between key exposures and outcomes were analyzed as risk ratios estimated using Poisson regression analyses with a robust variance. ^30^ Adjusted risk ratios were also estimated using Poisson regression accounting for other key exposures plus maternal age, BMI, estimated gestational age at enrollment, and HIV serostatus at enrollment. We then analyzed the association between continuous exposure variables and outcomes (gestational duration and birthweight centile by gestational age at birth) using linear regression.

Kaplan-Meier curves were plotted for time to delivery for participants with and without a history of prior PTB, short cervix, and twin gestation. We accounted for loss to follow-up by right censoring women at their last study visit (if before delivery) and compared survival between exposure groups by log-rank tests. We also used Cox regression to calculate the hazard of delivery between participants with and without prior PTB, short cervix, and twin gestation, adjusting for maternal age. The proportional-hazards assumption was tested based on Schoenfeld residuals. ^31^ Because of the inherent converging of survival curves in pregnancy at term, we restricted our models to the preterm period by administratively censoring all participants at 37 gestational weeks. ^32, 33^

All statistical analyses were performed with Stata version 14 (College Station, TX, USA) and SAS version 9.4 (Cary, NC, USA). 

## Results

**Figure 1.**
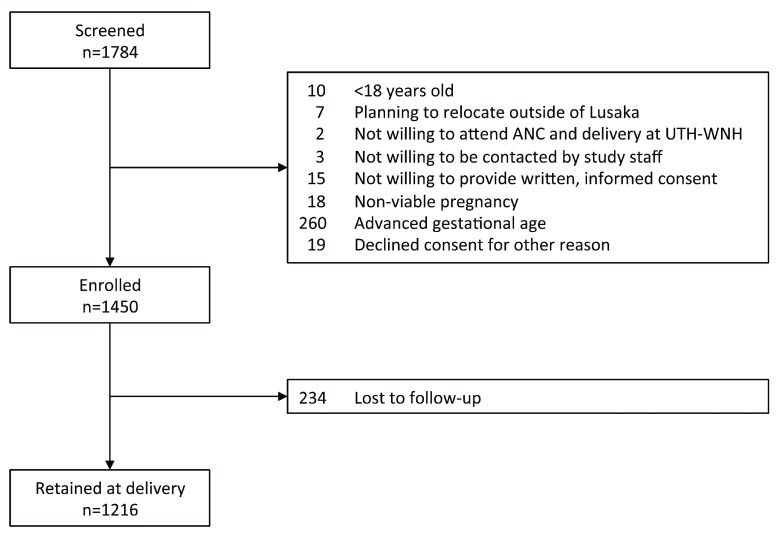
ZAPPS cohort participant flowchart. ANC, antenatal care; UTH-WNH, Women and Newborn Hospital of University Teaching Hospital.

From August 2015 to September 2017, 1784 pregnant women were screened and 1450 (81%) enrolled ( Figure 1). ^23^ The median age of enrolled participants was 27 (IQR: 23–32) ( Table 1). ^34^ Median estimated gestational age (EGA) at enrollment was 16 weeks; 30% (n=427/1450) were enrolled before 14 completed gestational weeks. Of 1042 (72%) participants who had been pregnant at least once in the past; 19% (n=194/1042) reported a prior miscarriage. Of 992 (68%) with a prior delivery, 41% (n=411) reported a prior PTB. On ultrasound exam, 3% (n=35/1175) had short cervix <25mm, and 3% (n=38/1450) had twin gestation. The prevalence of HIV seropositivity at enrollment was 24% (n=350/1447). Syphilis seropositivity was detected in 5% (70/1342).

**Table 1.** Baseline characteristics of ZAPPS cohort, N=1450.

Characteristic	Total enrolled N=1450		Retained at delivery visit N=1216 (83.9%)		Lost to follow-up N=234 (16.1%)		*p*
	N	Value % or Median (IQR) *	N	Value % or Median (IQR) *	N	Value % or Median (IQR) *	
Maternal age, years	1409	27 (23–32)	1192	27 (23–32)	217	24 (20–29)	<.001
<20	111	7.9	72	6.0	39	18.0	
20–34	1116	79.2	956	80.2	160	73.7	
≥35	182	12.9	164	13.8	18	8.3	
Missing	41		24		17		
Maternal education, years	1435	12 (9–12)	1204	12 (9–12)	231	9 (7–12)	<.001
None	26	1.8	19	1.6	7	3.0	
0–12 years	1225	85.4	1018	84.6	207	89.6	
≥12 years	184	12.8	167	13.9	17	7.4	
Missing	15		12		3		
Married or cohabiting	1202	83.7	1014	84.1	188	81.4	0.310
Missing	13		10		3		
Electricity in home	1302	90.6	1105	91.6	197	85.3	0.002
Missing	13		10		3		
Piped drinking water in home	1340	93.3	1123	93.2	217	93.9	0.678
Missing	14		11		3		
Toilet facilities in home							<.001
Flush or Pour	762	53.0	667	55.3	95	41.1	
Pit / Latrine / Other	675	47.0	539	44.7	136	58.9	
Missing	13		10		3		
Floor material in home							0.438
Natural / rudimentary	138	9.6	119	9.9	19	8.2	
Finished	1299	90.4	1087	90.1	212	91.8	
Missing	13		10		3		
Domestic violence in past year	71	5.0	58	4.9	13	5.6	0.641
Missing	28		26		2		
Smoking in pregnancy	8	0.6	7	0.6	1	0.4	0.768
Missing	24		23		1		
Alcohol use in pregnancy	124	8.7	106	8.9	18	7.7	0.563
Missing	25		24		1		
Maternal height at enrollment, cm	1368	156 (160–164)	1151	156 (160–165)	217	156 (160–164)	0.263
BMI at enrollment, kg/m ^2^	1366	23.6 (21.2–27.2)	1149	23.9 (21.4–27.6)	217	22.7 (20.7–25.5)	<.001
<18.5	71	5.2	56	4.9	15	6.9	
18.5–30.0	1103	80.8	919	80.0	184	84.8	
>30.0	192	14.1	174	15.1	18	8.3	
Missing	84		67		17		
Gravidity	1450	2 (1–4)	1042	2 (1–4)	408	2 (1–3)	0.003
Primigravida	408	28.1	321	26.4	87	37.2	0.001
Multigravida	1042	71.9	895	73.6	147	62.8	
Prior miscarriage, n=1042							0.933
Multigravida, no prior miscarriage	848	81.4	728	81.3	120	81.6	
Multigravida, ≥1 prior miscarriage	194	18.6	167	18.7	27	18.4	
Parity	1450	1 (0–2)	1216	1 (0–2)	234	1 (0–2)	0.004
Nulliparous	458	31.6	365	30.0	93	39.7	0.003
Parous	992	68.4	851	70.0	141	60.3	
Prior PTB, n=992							0.224
Parous, no prior PTB	581	58.6	505	59.3	76	53.9	
Parous, ≥1 prior PTB	411	41.4	346	40.7	65	46.1	
Prior stillbirth, n=992							0.401
Parous, no prior SB	780	86.1	672	86.5	108	83.7	
Parous, ≥1 prior SB	126	13.9	105	13.5	21	16.3	
Missing	86		74		12		
Short cervix < 2.5 cm	35	3.0	32	3.0	3	3.2	0.899
Missing	275		135		140		
Twin gestation	38	2.6	31	2.6	7	3.0	0.698
HIV positive at enrollment	350	24.2	304	25.0	46	19.7	0.084
Missing	3		2		1		
Syphilis reactive	70	5.2	63	5.6	7	3.1	0.142
Missing	108		93		15		
Hypertensive at enrollment ^^^	52	3.7	46	3.9	6	2.7	0.392
Missing	31		21		10		
Hemoglobin at enrollment, g/dL	1025	12 (11–13)	854	12 (11–13)	171	12 (11–13)	0.274
<10.5	140	13.7	123	14.4	17	9.9	
Missing	425		362		63		
Abnormal UA at enrollment *†*	69	5.0	55	4.8	14	6.3	0.343
Missing	79		67		12		
EGA at enrollment, weeks	1450	16.1 (13.3–18.3)	1216	16.0 (13.3–18.3)	234	16.3 (13.3–18.6)	0.421
<14	427	29.4	362	29.8	65	27.8	

BMI, body mass index; PTB, preterm birth; SB, stillbirth; UA, urinalysis; EGA, estimated gestational age; IQR, interquartile range.

* Not all columns sum to 100% due to rounding.

^^^ Defined as systolic blood pressure ≥ 140 and/or diastolic blood pressure ≥ 90.

*†* Defined as 1+ leukocyte esterase and/or + nitrites.

p values calculated by Wilcoxon rank sum or chi-square for continuous and categorical comparisons, respectively.

Of enrolled participants, 1216 (84%) were retained with delivery date ascertained. Compared to participants lost to follow-up, those retained at delivery were older (median: 27 versus 24 years, p<.001), had more years of education (median: 12 versus 9 years, p<.001), were more likely to have electricity (91% versus 85%, p=.002) and flush or pour toilet facilities at home (55% versus 41%, p<.001), had higher body mass index (23.9 versus 22.7 kg/m ^2^, p<.001), and had higher gravidity (74% versus 63% multigravid, p=.001) and parity (70% versus 60% parous, p=.004).

**Figure 2.**
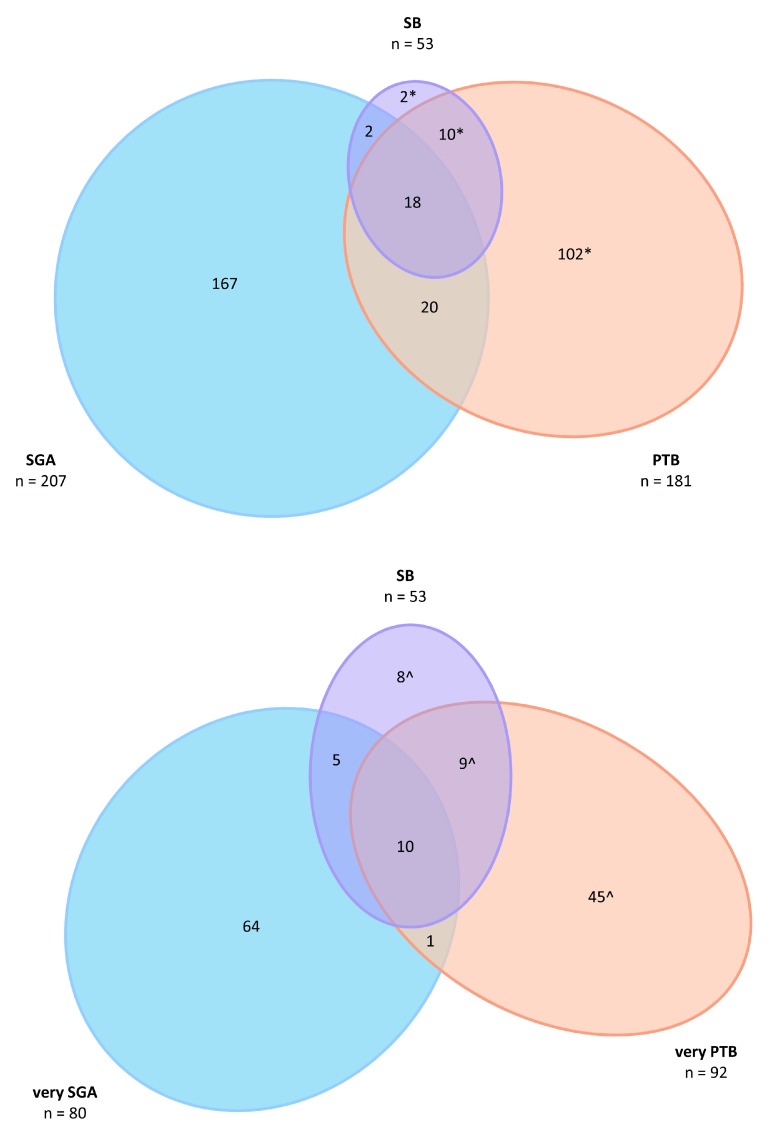
Preterm birth, very preterm birth, small for gestational age, very small for gestational age, and stillbirth among participants retained at delivery in ZAPPS cohort. Among ZAPPS cohort participants retained at delivery, 15% (181/1216) were preterm (PTB), 8% (92/1216) were very PTB, 18% (207/1159) were small for gestational age (SGA), 7% (80/1159) were very SGA, and 4% (53/1209) were stillborn (SB). *11 preterm births (^9 of which were very preterm), one term stillbirth, and 20 preterm stillbirths (^18 of which were very preterm) were either outside the gestational age threshold for INTERGROWTH-21 ^st^ calculation of SGA, ^28^ or were missing birthweight at delivery. Figures created with: EulerAPE. ^36^

Frequencies of our outcomes were as follows: 15% PTB (n=181/1216), 8% very PTB (n=92/1216), 18% SGA (n=207/1159), 7% very SGA (n=80/1159), and 4% SB (n=53/1209). Three participants (0.3%) experienced miscarriages before 16 weeks of gestation. Of the pregnancies that ended in SB, 44 (83%) were antepartum and 9 (17%) occurred intrapartum. Among 1159 deliveries within the EGA range for SGA calculation and with birthweight recorded, ^35^ 150 (13%) were PTB, 65 (6%) were very PTB, 207 (18%) were SGA, 80 (7%) were very SGA, and 32 (3%) were stillborn ( Figure 2).

**Figure 3.**
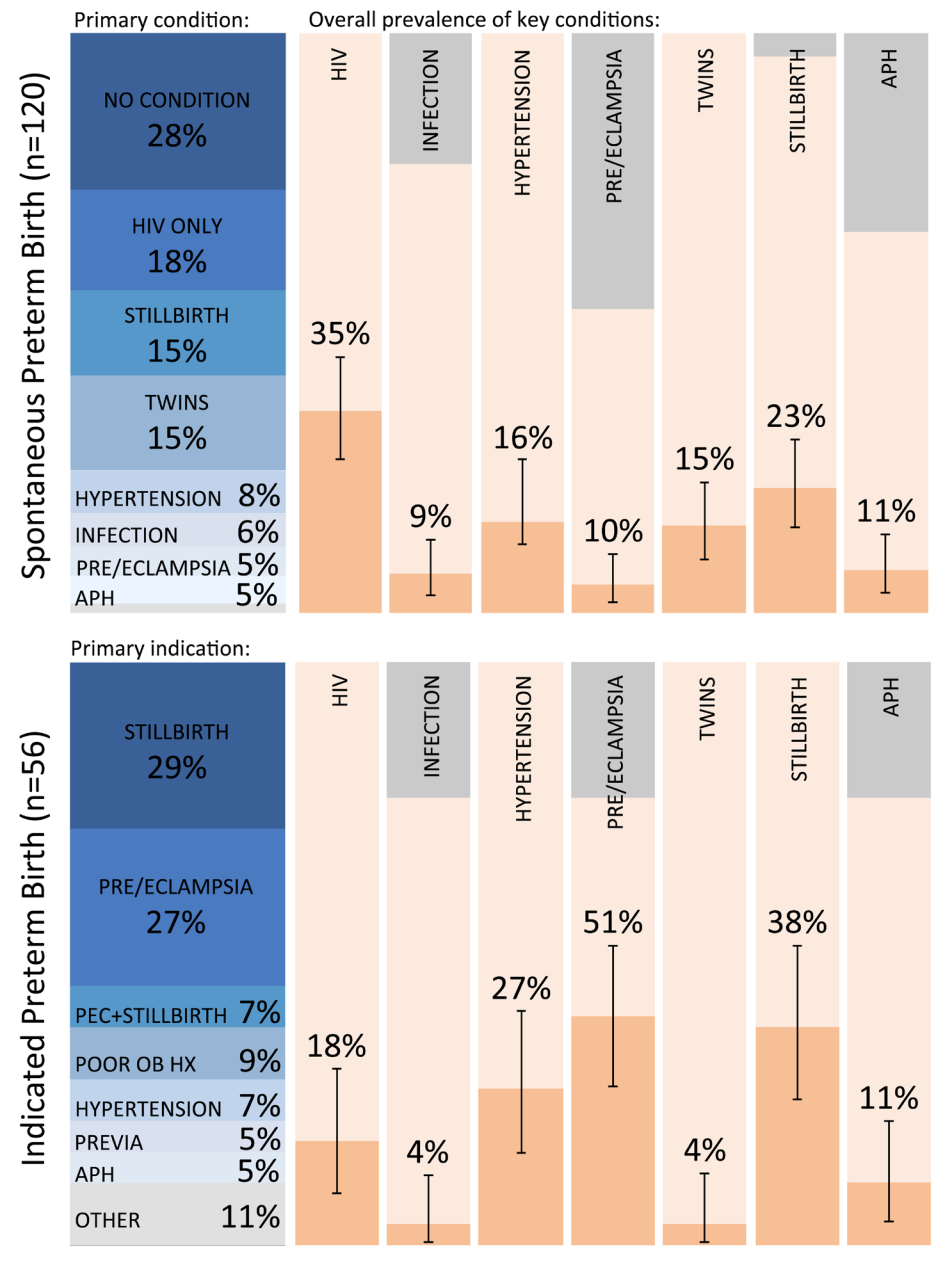
Parturition phenotypes among ZAPPS participants with preterm delivery. Of participants who underwent preterm delivery (n=181) in the ZAPPS cohort, 120 of them were spontaneous and 56 were indicated. This figure presents the frequencies of primary conditions present in spontaneous preterm deliveries, primary indications for indicated preterm deliveries, and the overall frequency with 95% confidence intervals of key conditions in each group. Gray bars represent missing values. APH, antepartum hemorrhage; OB HX, obstetrical history.

Of 181 total PTB, 120 (66%) occurred spontaneously, 56 (31%) were provider-initiated, and 5 (3%) could not be definitively classified ( Figure 3). The most common key conditions present in women with spontaneous PTB (n=120) were HIV infection (n=42, 35%), SB (n=26, 23%), hypertension alone (n=22; 18%), and twin gestation (n=18, 15%); 33 (28%) had no key condition identified. Most provider-initiated preterm deliveries were indicated for SB (n=16, 29%), preeclampsia or eclampsia (n=15, 27%) or hypertension alone (n=4, 7%), or both SB and preeclampsia (n=4, 7%). We identified major phenotypic clusters of PTB, spontaneous PTB, and provider-initiated PTB by presence of maternal, fetal, and/or placental conditions ( Table 2).

**Table 2.** Phenotypes of preterm birth in ZAPPS cohort, N=181.

	All preterm birth N (%) *		Spontaneous N (%) †		Provider-initiated N (%) †	
**All preterm**	181	100	120	68	56	32
**Phenotypic clusters**						
No significant clinical conditions	41	23	33	87	5	13
Maternal condition(s) only	60	33	40	67	20	33
Fetal condition(s) only	27	15	21	81	5	19
Placental condition(s) only	4	2	1	25	3	75
Maternal and fetal conditions	37	20	17	47	19	53
Maternal and placental conditions	6	3	3	50	3	50
Fetal and placental conditions	1	1	1	100	0	0
Maternal, fetal, and placental conditions	5	3	4	80	1	20
**Significant maternal conditions**	108	60	64	60	43	40
HIV infection	53	29	42	81	10	19
Urinary tract infection, n=41	9	22	7	78	2	22
Clinical chorioamnionitis, n=120	1	1	1	100	0	0
Diabetes (mellitus or gestational), n=179	5	3	1	20	4	80
Hypertension	34	19	19	56	15	44
Preeclampsia, n=107	23	21	6	26	17	74
Eclampsia, n=125	5	4	0	0	5	100
**Significant fetal conditions**	70	39	43	63	25	37
Twin gestation	21	12	18	90	2	10
Stillbirth, n=176	48	27	26	55	21	45
Fetal growth restriction	2	1	0	0	2	100
Fetal distress	1	1	0	0	1	100
Polyhydramnios	1	1	0	0	1	100
Oligohydramnios	1	1	0	0	1	100
**Significant placental conditions**	16	9	9	56	7	44
Placental abruption	15	8	9	60	6	40
Placenta previa	4	2	0	0	4	100

* column percent.

† row percent.

**Figure 4.**
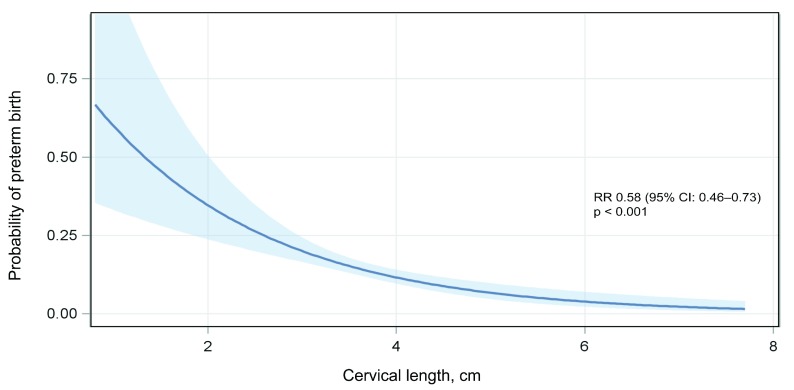
Predicted probability of preterm birth <37 weeks by mid-trimester cervical length. Among ZAPPS cohort participants with a cervical length measured by ultrasound in the second trimester (n=1081), the probability of preterm birth <37 weeks decreased with increasing cervical length. PTB, preterm birth; RR, relative risk; CI, confidence interval.

Maternal age ≥35, prior PTB, short cervix, twin gestation, antenatal hypertension, and EGA at enrollment <14 weeks were associated with PTB ( Table 3). Overall, these associations were stable or strengthened when restricting the outcome to spontaneous PTB and to very PTB ( Table 4). Although associated with PTB, antenatal hypertension did not significantly predict spontaneous phenotypes of PTB. Maternal height was not significantly associated with PTB or very PTB in univariate analyses. In multivariable regression models adjusting for maternal age, BMI, EGA at enrollment, and HIV status at enrollment, participants with prior PTB (aRR 1.88; 95% CI 1.32–2.68), short cervix (aRR 2.62; 95% CI 1.68–4.09), twin gestation (aRR 5.22; 95% CI 3.67–7.43), and antenatal hypertension (aRR 2.04; 95% CI 1.43–2.91) had increased risk of PTB ( Table 2). The associations between the exposures of prior PTB, short cervix, and twin gestation with PTB were stable or strengthened when restricting the outcome to spontaneous phenotypes and very PTB ( Table 4). The risk of PTB decreased with increasing cervical length (RR 0.58 per cm; 95% CI 0.46–0.73) ( Figure 4). In multiple linear regression of continuous exposure variables, gestational duration was associated with number of prior PTB (coeff -5.11 days; 95% CI -6.42 to -3.79), cervical length (coeff 6.49 days; 95% CI 4.18–8.81), and EGA at enrollment (0.70 days; 0.28–1.12) ( Table 6).

**Table 3.** Risk of adverse birth outcomes among ZAPPS participants retained at delivery, n=1213.

Exposure	Preterm birth 16 to <37 weeks							Spontaneous preterm birth 16 to <37 weeks							Small for gestational age							Stillbirth						
	n	events	%	RR	95% CI	aRR *	95% CI	n	events	%	RR	95% CI	aRR *	95% CI	n	events	%	RR	95% CI	aRR ‡	95% CI	n	events	%	RR	95% CI	aRR ‡	95% CI
Age at enrollment, years																												
<20	72	5	6.9	1.00				70	3	4.3	1.00				70	13	18.6	1.00				72	0	0.0	-			
20–34	953	144	15.1	2.18	0.92– 5.14			950	103	10.8	2.53	0.82– 7.77			911	157	17.2	0.93	0.56– 1.55			954	39	4.1	1.00			
≥35	164	31	18.9	2.72	1.10– 6.72			164	13	7.9	1.85	0.54– 6.29			155	33	21.3	1.15	0.64– 2.04			159	14	8.8	2.15	1.20– 3.88		
BMI at enrollment, kg/m ^2^																												
<18.5	56	12	21.4	1.00				56	10	17.9	1.00				53	10	18.9	1.00				55	1	1.8	1.00			
18.5–30.0	916	136	14.9	0.69	0.41– 1.17			912	90	9.9	0.55	0.30– 1.00			876	172	19.6	1.04	0.59– 1.85			913	41	4.5	2.47	0.35– 17.6		
>30.0	174	20	11.5	0.54	0.28– 1.03			173	10	5.8	0.32	0.14– 0.74			168	18	10.7	0.57	0.28– 1.15			173	7	4.1	2.23	0.28– 17.7		
Prior PTB																												
Nulliparous	364	40	11.0	1.13	0.76– 1.68	0.93	0.60– 1.43	363	25	6.9	1.23	0.73– 2.08	0.86	0.47– 1.55	345	73	21.2	1.42	1.06– 1.91	1.36	0.98– 1.87	363	8	2.2	0.62	0.27– 1.40	0.95	0.36– 2.50
Parous, no prior PTB	504	49	9.7	1.00		1.00		502	28	5.6	1.00		1.00		491	73	14.9	1.00		1.00		503	18	3.6	1.00		1.00	
Parous, ≥1 prior PTB	345	92	26.7	2.74	1.99– 3.77	1.88	1.32– 2.68	343	67	19.5	3.5	2.30– 5.33	2.62	1.69– 4.06	323	61	18.9	1.27	0.93– 1.73	1.12	0.82– 1.54	343	27	7.9	2.20	1.23– 3.93	1.63	0.78– 3.40
Cervical length																												
≥2.5cm	1049	137	13.1	1.00		1.00		1045	91	8.7	1.00		1.00		1021	181	17.7	1.00				1046	35	3.4	1.00		1.00	
<2.5cm	32	16	50.0	3.83	2.62– 5.60	2.62	1.68– 4.09	32	9	28.1	3.23	1.79– 5.81	1.95	1.01– 3.77	31	9	29.0	1.64	0.93– 2.89			32	6	18.8	5.60	2.54– 12.4	6.42	2.56– 16.1
Gestation																												
Single	1182	160	13.5	1.00		1.00		1178	102	8.7	1.00		1.00		1128	191	16.9	1.00		1.00		1177	51	4.3	1.00			
Twin	31	21	67.7	5.00	3.77– 6.64	5.22	3.67– 7.43	30	18	60.0	6.93	4.90– 9.80	7.86	5.37– 11.5	31	16	51.6	3.05	2.12– 4.39	2.75	1.81– 4.18	31	2	6.5	1.49	0.38– 5.85		
HIV serostatus at enrollment																												
Negative	908	128	14.1	1.00		1.00		904	78	8.6	1.00		1.00		871	152	17.5	1.00		1.00		906	37	4.1	1.00		1.00	
Positive	303	53	17.5	1.24	0.93– 1.66	1.17	0.85– 1.62	302	42	13.9	1.61	1.13– 2.29	1.36	0.91– 2.03	286	55	19.2	1.10	0.83– 1.46	1.09	0.80– 1.47	301	16	5.3	1.30	0.73– 2.31	1.29	0.65– 2.56
Syphilis																												
Non-reactive	1057	159	15.0	1.00				1053	109	10.4	1.00				1006	184	18.3	1.00				1053	41	3.9	1.00		1.00	
Reactive	63	9	14.3	0.95	0.51– 1.77			63	3	4.8	0.46	0.15– 1.41			63	13	20.6	1.13	0.68– 1.86			63	6	9.5	2.45	1.08– 5.55	2.34	0.91– 6.04
Blood pressure during pregnancy																												
Normotensive	1072	145	13.5	1.00		1.00		1067	106	9.9	1.00				1025	173	16.9	1.00		1.00		1068	42	3.9	1.00		1.00	
Hypertensive ‡	141	36	25.5	1.89	1.37– 2.60	2.04	1.43– 2.91	141	14	9.9	1.00	0.59– 1.70			134	34	25.4	1.50	1.09– 2.07	1.62	1.16– 2.26	141	11	7.8	1.98	1.05– 3.76	1.83	0.84– 3.96
Hemoglobin at enrollment																												
≥10.5 g/dL	730	111	15.2	1.00				727	70	9.6	1.00				700	125	17.9	1.00				726	30	4.1	1.19	0.51– 2.80		
<10.5 g/dL	121	21	17.4	1.14	0.75– 1.75			121	15	12.4	1.29	0.76– 2.17			113	20	17.7	0.99	0.65– 1.52			122	6	4.9	1.00			
UA during pregnancy																												
Normal	907	125	13.8	1.00				903	82	9.1	1.00				880	168	19.1	1.00				903	32	3.5	1.00			
Abnormal ^^^	189	31	16.4	1.19	0.83– 1.71			189	20	10.6	1.17	0.73– 1.85			182	26	14.3	0.75	0.51– 1.10			191	6	3.1	0.89	0.38– 2.09		
EGA at enrollment, weeks																												
<14	360	72	20.0	1.00				358	45	12.6	1.00				333	62	18.6	1.00				357	20	5.6	1.00			
≥14	853	109	12.8	0.64	0.49– 0.84			850	75	8.8	0.70	0.50– 0.99			826	145	17.6	0.94	0.72– 1.23			851	33	3.9	0.69	0.40– 1.19		

BMI, body mass index; PTB, preterm birth; UA, urinalysis; EGA, estimated gestational age; RR, relative risk; CI, confidence interval; aRR, adjusted risk ratio

* Risk ratios calculated via Poisson regression with robust error variance. Multivariable model estimates of adjusted risk ratios include other exposure variables listed and all models adjusted for: maternal age, maternal BMI, and EGA at enrollment as continuous variables.

‡ Defined as systolic blood pressure ≥ 140 and/or diastolic blood pressure ≥ 90 at enrollment or at any follow-up ANC visit.

^^^ Defined as 1+ leukocyte esterase and/or + nitrites.

**Table 4.** Risk of severe adverse birth outcomes in ZAPPS participants retained at delivery, n=1213.

Exposure	Very preterm birth 16 to <34 weeks						Spontaneous very preterm birth 16 to <34 weeks								Very small for gestational age						
	n	events	%	RR	95% CI	aRR *	95% CI	n	events	%	RR	95% CI	aRR *	95% CI	n	events	%	RR	95% CI	aRR *	95% CI
Age at enrollment, years																					
<20	72	3	4.2	1.00				71	2	2.8	1.00				70	7	10.0	1.00			
20–34	953	75	7.9	1.89	0.61–5.84			950	50	5.3	1.87	0.46–7.53			913	55	6.0	1.66	0.79–3.51		
≥35	164	13	7.9	1.90	0.56–6.48			164	6	3.7	1.30	0.27–6.28			155	17	11.0	1.82	1.09–3.05		
BMI at enrollment, kg/m ^2^																					
<18.5	56	4	7.1	1.00				56	3	5.4	1.00				53	8	15.1	1.00			
18.5–30.0	916	72	7.9	1.10	0.42–2.90			913	46	5.0	0.94	0.30–2.93			879	60	6.8	0.45	0.23–0.90		
>30.0	174	9	5.2	0.72	0.23–2.27			173	5	2.9	0.54	0.13–2.19			167	10	6.0	0.40	0.17–0.95		
Prior PTB																					
Nulliparous	364	22	6.0	1.45	0.81–2.60	1.10	0.55–2.21	364	14	3.9	1.61	0.75–3.44	0.88	0.34–2.31	345	32	9.3	1.98	1.18–3.32	1.92	1.12–3.32
Parous, no prior PTB	504	21	4.2	1.00		1.00		502	12	2.4	1.00		1.00		491	23	4.7	1.00		1.00	
Parous, ≥1 prior PTB	345	49	14.2	3.41	2.08–5.58	2.27	1.28–4.04	343	33	9.6	4.02	2.11–7.68	2.89	1.39–5.98	323	25	7.7	1.65	0.95–2.86	1.39	0.76–2.53
Cervical length																					
≥2.5cm	1049	63	6.0	1.00		1.00		1046	39	3.7	1.00		1.00		1022	68	6.7	1.00		1.00	
<2.5cm	32	12	37.5	6.24	3.76–10.4	3.97	2.15–7.33	32	7	21.9	5.87	2.84–12.1	3.19	1.35–7.55	31	5	16.1	2.42	1.05–5.59	2.06	0.88–4.82
Gestation																					
Single	1182	81	6.9	1.00		1.00		1179	51	4.3	1.00		1.00		1130	74	6.6	1.00		1.00	
Twin	31	11	35.5	5.18	3.08–8.70	5.18	2.75–9.77	30	8	26.7	6.16	3.21–11.8	7.53	3.58–15.9	31	6	19.4	2.96	1.39–6.27	2.71	1.12–6.57
HIV serostatus at enrollment																					
Negative	908	67	7.4	1.00		1.00		905	41	4.5	1.00		1.00		871	61	7.0	1.00		1.00	
Positive	303	25	8.3	1.12	0.72–1.74	1.20	0.71–2.01	302	18	6.0	1.32	0.77–2.26	1.35	0.68–2.66	286	19	6.6	0.95	0.58–1.56	0.86	0.48–1.53
Syphilis																					
Non-reactive	1057	81	7.7	1.00				1054	53	5.0	1.00				1009	71	7.0	1.00			
Reactive	63	5	7.9	1.04	0.44–2.46			63	2	3.2	0.63	0.16–2.53			63	6	9.5	1.35	0.61–2.99		
Blood pressure during pregnancy																					
Normotensive	1072	78	7.3	1.00				1068	55	5.2	1.00				1025	65	6.3	1.00		1.00	
Hypertensive ‡	141	14	9.9	1.36	0.79–2.34			141	4	2.8	0.55	0.20–1.50			134	15	11.2	1.77	1.04–3.00	1.68	0.92–3.06
Hemoglobin at enrollment																					
≥10.5 g/dL	730	60	8.2	1.00				727	35	4.8	1.00				703	46	6.5	1.00			
<10.5 g/dL	121	7	5.8	0.70	0.33–1.50			121	5	4.1	0.86	0.34–2.15			113	5	4.4	0.68	0.27–1.67		
UA during pregnancy																					
Normal	907	59	6.5	1.00				904	37	4.1	1.00				881	68	7.7	1.00			
Abnormal ^^^	189	14	7.4	1.14	0.65–2.00			189	7	3.7	0.81	0.41–2.00			183	10	5.5	0.71	0.37–1.35		
EGA at enrollment, weeks																					
<14	360	39	10.8	1.00				358	26	7.3	1.00				333	25	7.5	1.00			
≥14	853	53	6.2	0.57	0.39–0.85			851	33	3.9	0.92	0.86–0.99			828	55	6.6	0.88	0.56–1.40		

BMI, body mass index; PTB, preterm birth; UA, urinalysis; EGA, estimated gestational age; RR, relative risk; CI, confidence interval; aRR, adjusted risk ratio

* Risk ratios calculated via Poisson regression with robust error variance. Multivariable models include other exposure variables listed and adjusted for: maternal age, maternal BMI, and EGA at enrollment.

‡ Defined as systolic blood pressure ≥ 140 and/or diastolic blood pressure ≥ 90 at enrollment or at any follow-up visit.

^^^ Defined as 1+ leukocyte esterase and/or + nitrites.

Nulliparity, twin gestation, and antenatal hypertension were each associated with SGA in univariate analysis, and older age, low BMI, nulliparity, short cervix, twin gestation, and antenatal hypertension were associated with very SGA. In multivariable analysis, twin gestation (aRR 2.75; 95% CI 1.81–4.18) and antenatal hypertension (aRR 1.62; 95% CI 1.16–2.26) were associated with an increased risk of SGA; nulliparity was marginally associated with SGA (aRR 1.36; 95% CI 0.98–1.87). Nulliparity (aRR 1.92; 95% CI 1.12–3.32) and twin gestation (aRR 2.71, 95% CI 1.12–6.57) were associated with very SGA. Maternal height was not associated with SGA or very SGA in univariate analyses of categorical outcomes, but was associated with mean centile of birthweight in adjusted linear regression (coeff 0.32; 95% CI 0.04,0.59), along with maternal weight (coeff 0.34; 95% CI 0.21,0.47), and EGA at enrollment (coeff -0.75; 95% CI -1.25 to -0.25) ( Table 6).

Finally, older maternal age, prior PTB, short cervix, syphilis seropositivity, and antenatal hypertension were individually associated with an elevated risk of SB ( Table 3). In multivariable analysis, short cervix predicted SB (aRR 6.42; 95% CI 2.56–16.1), while syphilis was only marginally associated (aRR 2.34; 95% CI 0.91–6.04).

**Figure 5.**
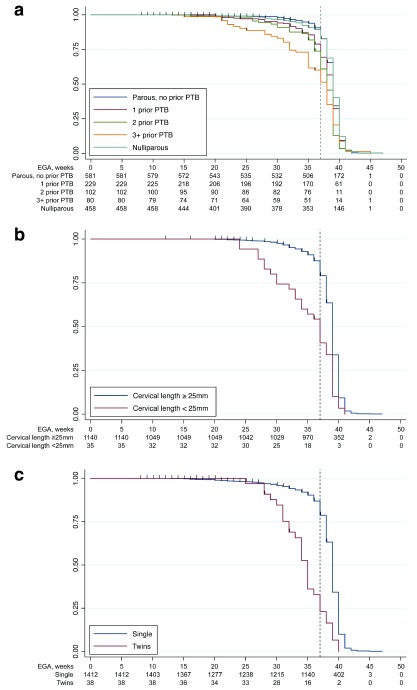
Kaplan-Meier survival curves by ( **a**) prior preterm birth, ( **b**) short cervix (<25mm), and ( **c**) twin gestation. Survival curves are presented for participants with increasing numbers of prior preterm birth, those with cervical length <25mm compared to ≥25 mm, and those with twin compared to singleton gestation. The dashed vertical line represents a gestational age of 37 weeks, the threshold for preterm versus term delivery. EGA, estimated gestational age; PTB, preterm birth.

Elevated risks of PTB among women with prior PTB, short cervix, and twin gestation were supported by survival analyses, with log-rank tests of association demonstrating significant differences between groups of each variable ( Figure 5; Table 5). In proportional hazards models adjusted for maternal age at enrollment, participants with prior PTB, short cervix, and twin gestation had significantly higher hazards of delivering before 37 gestational weeks compared to parous women with no prior PTB, women with cervical lengths ≥25mm, or with single gestations. Participants with increasing numbers of prior preterm births demonstrated increasing hazard ratios of delivering preterm.

**Table 5.** Log-rank and Cox proportional hazards regression with test of proportionality assumption for prior preterm birth, short cervical length, and twin gestation.

	Log-rank	Cox proportional hazards			Schoenfeld residual test		
	*p*	HR *	95% CI	*p*	*rho*	*Χ* ^2^	*p*
Prior preterm birth					*global*	3.23	0.66
Parous, no prior	<.001 ^^^	*ref*			*ref*		
Parous, 1 prior		2.21	1.47– 3.33	<.001	-0.01	0.01	0.92
Parous, 2 prior		2.79	1.70– 4.58	<.001	0.00	0.00	0.95
Parous, 3+ prior		4.70	2.94– 7.53	<.001	-0.11	2.33	0.13
Nulliparous	-	1.16	0.75– 1.78	0.51	-0.06	0.80	0.37
Cervical length					*global*	4.42	0.11
≥ 25mm	<.001	*ref*			*ref*		
<25mm		5.19	3.09– 8.74	<.001	-0.15	3.26	0.07
Gestation					*global*	3.19	0.20
Singleton	<.001	*ref*			*ref*		
Twin		6.70	4.25– 10.60	<.001	0.13	3.19	0.07

HR, hazards ratio; CI, confidence interval.

* Each proportional hazards model adjusted for maternal age at enrollment.

^^^ log-rank of trend, excluding nulliparas.

**Table 6.** Association between continuous exposures by gestational duration and birthweight centile.

Exposure	Gestational duration, days				Birthweight centile for gestational age			
	coeff	95% CI	adjusted	95% CI	coeff	95% CI	adjusted	95% CI
Age at enrollment, years	-0.18	-0.44,0.08			0.30	0.01,0.60	-0.02	-0.32,0.29
Maternal height at enrollment, m	-0.05	-0.28,0.17			0.63	0.37,0.88	0.32	0.04,0.59
Maternal weight at enrollment, kg	-0.01	-0.11,0.09			0.41	0.30,0.53	0.34	0.21,0.47
BMI at enrollment, kg/m ^2^	0.09	-0.20,0.37			0.84	0.51,1.17		
Number of prior PTB	-6.54	-8.07,-5.00	-5.11	-6.42,-3.79	-1.94	-3.80,-0.07		
Cervical length	6.93	5.05,8.82	6.49	4.18,8.81	0.87	-1.84,3.58		
Systolic blood pressure at enrollment	-0.11	-0.22,0.01			-0.05	-0.18,0.08		
Diastolic blood pressure at enrollment	-0.17	-0.32,-0.02			-0.01	-0.18,0.17		
Hemoglobin at enrollment	0.45	-0.59,1.48			1.17	-0.01,2.34		
EGA at enrollment, weeks	1.12	0.70,1.54	0.70	0.28,1.12	-0.77	-1.26,-0.29	-0.75	-1.25,-0.25

Coefficients and confidence intervals calculated by linear regression. Adjusted coefficients calculated in multivariable models that included other exposure variables with estimates shown.

PTB, preterm birth; EGA, estimated gestational age; coeff, coefficient; CI, confidence interval.

## Discussion

We present the primary results of the ZAPPS pregnancy cohort, established to evaluate the risk factors associated with adverse birth outcomes in Lusaka, Zambia. This study was notable for enrollment of pregnant women at early presentation to antenatal care, gestational age determination by early ultrasound, universal cervical length screening, comprehensive and uniform antenatal and postpartum care, and clinical phenotyping of birth outcomes. Our analyses revealed strong risks of prior preterm birth, short mid-trimester cervical length, and twin gestation on incident preterm birth, and these risks were supported by analyses of pregnancy ‘survival’ to term. We also report increased risks of small-for-gestational-age infants among nulliparous women and women with twin gestation, and of stillbirth among those with short cervix.

The proportion of gravidas who deliver before term varies significantly across individual studies and national estimates in sub-Saharan Africa. The most recent global report estimated a PTB rate of 8% in Europe and 11% in North America, compared to 12% across sub-Saharan Africa and 12% in Zambia specifically, where the rate was based on modeled regional estimates instead of national data. ^6^ In contrast, a census accounting of 237,219 public sector births over 6 years in Lusaka — where the vast majority of pregnancies are dated by last menstrual period — classified 46% of singleton deliveries as preterm. ^37, 38^ In Zambia, obstetrical ultrasound is rare and the reliance on maternal recall of LMP alone substantially over-estimates preterm birth rates, ^17, 39^ an inaccuracy that worsens with later presentation to care. ^21^ We report PTB based on ultrasound gestational age dating and prospectively ascertained delivery outcomes such that our data are likely more accurate than reports that rely on LMP recall or regional models.

Inconsistent global PTB definitions hinder inter-regional comparisons of rates and risk factors, and we acknowledge that gestational age boundaries are somewhat arbitrary. We chose a lower gestational age limit of 16 weeks because of evidence of similar etiological risk factors between preterm births occurring as early as 16 weeks and those that occur later in pregnancy ^12, 40^. In addition, we included preterm stillbirths in our definition of PTB since excluding stillbirths that occur in the process of parturition would falsely lower the rate of PTB. In a sensitivity analysis that excluded all stillbirths from PTB outcomes, the PTB incidence was modestly reduced. However, risk estimates calculated in regression models remained stable, which supports evidence that risk factors for live and stillborn PTB demonstrate substantial overlap ^40^.

The distinction of preterm parturition as spontaneously occurring versus provider-initiated is important but rarely reported from national surveillance or clinical research data in low-resource settings. Deliveries that are preceded by spontaneous labor or membrane rupture are phenotypically distinct from those that are induced medically or surgically for complications such as preeclampsia, antepartum fetal demise, or other maternal or fetal conditions. ^12, 41– 44^ Further classification based on primary conditions present in spontaneous PTB and the primary indications for provider-initiated PTB is based on a standardized rubric proposed to elucidate phenotypic clusters of PTB ^12^. An understanding of prevailing phenotypes can direct research, policy, and preventive interventions towards regional and population-specific needs. ^13, 45, 46^ While our cohort is limited by a small number of PTB events (n=181), we were able to classify nearly all (i.e., 97%) as either spontaneous or provider-initiated, and to identify the primary complications and phenotypic characteristics of each. Further granularity and generalizability of PTB classification requires a larger sample size, signaling a need for future high-quality obstetrical research on a greater scale.

As with PTB classification, identifying infants born SGA requires accurate gestational age estimation, which can be at best imprecise, and at worst biased, when based solely on LMP. ^21^ The incidence of SGA in our cohort (18%) was modestly higher than regional estimates of SGA in sub-Saharan Africa of 16%, ^5^ and compared to a recent estimate in Zambia of 13%, which was modeled from published rates in other neighboring countries because of scarcity of data from Zambia itself. ^5^ In comparison to the ZAPPS cohort, in which older maternal age, low BMI, nulliparity, twin gestation, and antenatal hypertension predicted either SGA or very SGA, a study among over 19,000 singletons in Tanzania identified *younger* maternal age, height, and nulliparity as strong risk factors for SGA. ^47^ The WHO Multi-country Survey on Maternal and Newborn Health found nulliparity and hypertensive disorders to indicate higher risk of preterm SGA and hypertensive disorders, sociodemographic factors, and anemia to predict term SGA. ^48^ With a much smaller sample size and fewer outcomes compared to these two studies, we were not able to differentiate our outcome by preterm vs. term SGA due to low statistical precision for stratified associations with key risk factors. Due to this low precision, we are not able to discern whether or not SGA outcomes were modified by gestational age at delivery. However, both of these studies relied on reported LMP to estimate gestational age at delivery, which itself may have introduced error. Whether growth restriction is a distinct pathological process before 37 weeks compared to after 37 weeks is unclear. Finally, while the INTERGROWTH-21 ^st^ Project ^49^ intended to define universal fetal growth and newborn weight standards derived from an extensive multi-ethnic sample of women with adequate antenatal care and nutrition, its widespread use over ethnicity-specific or customized standards has been disputed. ^43, 50– 56^ Despite this, we chose to define SGA in our cohort based on INTERGROWTH-21 ^st^ standards since local standards that include all pregnancies affected by undernutrition and/or pregnancy comorbidities tend to identify only the severest 10% of cases by definition.

Stillbirth, a composite outcome comprising antepartum and intrapartum fetal death, is particularly understudied in low-resource settings. Compared to developed regions with a stillbirth rate of 3.4 per 1000 total births, the rate in sub-Saharan Africa is estimated as 28.7 per 1000, while 4% of our cohort delivered stillbirths. ^3^ The true global burden of stillbirth and its underlying causes are poorly classified due to inconsistent fetal viability limits and imperfect classification of neonatal death versus stillbirth, limited resources for case investigations, under-reporting of home births that result in perinatal death, and inadequate national and regional reporting of identified cases. ^3^ Indeed, recent global and regional estimates of stillbirth included just 17% of its datapoints from sub-Saharan Africa and south Asia, regions that bear 77% of the global burden. ^3^ Data from the recent Zambia Demographic and Health Survey reported a rate of stillbirth, defined as fetal death over 7 months’ gestation, as 1.3% among 13,563 births reported, with equal rates outside Lusaka province as within. ^57^ This is similar to estimates from a Global Network study in Zambia, in which 2% of women enrolled delivered stillbirths. ^9^ The higher proportion of deliveries that resulted in stillbirth in the ZAPPS cohort, at least partly attributable to a broader gestational age range, was reflected in the ZEPRS database, in which 6% of 66,395 deliveries at UTH resulted in stillbirth. ^38^ However, over half of stillbirths in ZEPRS and 67% in a Global Network study in Zambia were classified as intrapartum, compared to less than 20% in the ZAPPS cohort. These disparities may result from differential classification; it is standard practice outside of our study to classify stillbirths solely by neonatal skin maceration at delivery, particularly in the absence of but even despite the presence of documented fetal heart activity during labor. ^9^ Indeed, previous studies have demonstrated that reliance on observed skin maceration alone can over-estimate stillbirth proportions attributable to the intrapartum period. ^58^

This study has several limitations, many of which have been noted previously. ^23^ First, 16% of participants were lost to follow-up. While this is commensurate with other longitudinal pregnancy cohort studies in the region, ^59, 60^ error may be introduced if outcomes are not missing at random. ^61^ Women lost to follow-up were younger, more likely to be primigravida and nulliparous, had lower BMIs, and had multiple lower measures of socioeconomic status; many of these characteristics were risk factors for at least one adverse outcome. Further, 250 (21%) of the retained participants either did not deliver at the study hospital or delivered at a time when ZAPPS staff were not present, requiring delivery outcomes to be ascertained by record review and/or participant report (it is worth noting that we found no difference in frequencies of outcomes between deliveries attended by ZAPPS staff versus those that were not; see *Underlying data*). Second, our data have noted missingness of key antenatal test results at baseline (i.e., hemoglobin, syphilis, and urinalysis) because tests were not routinely repeated nor results recorded in our database if performed at the recruitment clinic before enrollment. Of these test results, only syphilis was associated with an outcome (stillbirth), but we cannot determine with certainty whether missingness introduced bias or simply reduced statistical power. Third, while the ZAPPS study recruits from several surrounding primary clinics, it is based at a tertiary referral hospital and many of our participants were drawn from this higher-risk pool. We note high prevalence of prior PTB, miscarriage, and stillbirth, and high HIV and syphilis seropositivity, which may have resulted from self-selection of high-risk women into a cohort study investigating adverse birth outcomes. It is likely that this resulted in an over-representation of outcomes, but less likely to have also introduced a biased association with identified risk factors.

In summary, the ZAPPS cohort study demonstrates high prevalence of antenatal comorbidities and identifies a number of factors associated with increased risks of preterm birth, small-for-gestational-age infants, and stillbirth. This is the first study of its kind to be conducted in Zambia, and one of the largest on the African continent. An understanding of the true global scope of adverse birth outcomes will require consistent definitions, meticulous ascertainment, and systematic reporting that has eluded those settings where the burden of these outcomes is highest. In the absence of sophisticated registry infrastructure, large pregnancy cohort studies may be able to approximate regional incidence estimates and can provide important data to stratify and direct care for pregnancies at highest risk. Future sub-studies using data and stored biological specimens from the ZAPPS cohort will aim to identify underlying biological mechanisms, causal pathways, and appropriate interventions for the accurate prediction and prevention of adverse birth outcomes in Zambia and worldwide.

## Data availability

### Underlying data

Open Science Framework: Zambian Preterm Birth Prevention Study (ZAPPS) – Outcomes. https://doi.org/10.17605/OSF.IO/WT6Q8 ^34^

This project contains the following underlying data:

- Z1A minimum dataset 2019-06-30.csv (underlying data for all participants)
- Z1A Codebook 2019-06-30.rtf (codebook for the variables within the dataset)

Data are available under the terms of the Creative Commons Attribution 4.0 International license (CC-BY 4.0).
