# Supplementary material for: Adverse birth outcomes and their clinical phenotypes in an urban Zambian cohort
Source: Gates Open Res. 2020 Jan 24;3:1533. Originally published 2019 Sep 4. [Version 2] doi: 10.12688/gatesopenres.13046.2 (PMC7047437; doi:10.12688/gatesopenres.13046.2)
Supplement: Peer review history

# Peer review: Reviewer response for version 2

**DOI:** 10.21956/gatesopenres.14236.r28503

**Published:** 2020-02-26

**Authors:** Shinjini Bhatnagar, Ramachandran Thiruvengadam

**Affiliations:** 1 Maternal and Child Health program, Translational Health Science and Technology Institute, Faridabad, Haryana, India; 2 Translational Health Science and Technology Institute, Faridabad, India

I have no further comments to make.

We confirm that we have read this submission and believe that we have an appropriate level of expertise to confirm that it is of an acceptable scientific standard.

# Peer review: Reviewer response for version 1

**DOI:** 10.21956/gatesopenres.14168.r28082

**Published:** 2019-11-06

**Authors:** Shinjini Bhatnagar

**Affiliations:** 1 Maternal and Child Health program, Translational Health Science and Technology Institute, Faridabad, Haryana, India

In this manuscript, the authors report the rates of adverse birth outcomes such as preterm birth, stillbirth and small for gestational age from a well-characterized cohort of 1450 pregnant women from Zambia. This is a very important article, providing robust data from a low-middle income country like Zambia in the domain of maternal and child health. 

 The emphasis on accuracy and precision of gestational age estimation which is a cornerstone in preterm birth research is praise-worthy. The data is well represented and the statistical analyses are appropriate.

***Comments:***

1. Preterm birth has been defined as birth between 16 0/7 and 36 6/7 gestational weeks in this study. The definition seems to include stillbirths (the proportion of stillbirth among PTB is reported in paragraph 4 of the results section), which is in variance with the convention of reporting preterm birth among live-born babies. What is the rationale behind this choice? Does such a change in definition influence the preterm birth rate in this study population? If so, how much? 
2. The lower limit of gestational age for defining preterm birth is taken as 16 0/7 weeks in this study. What was the rationale behind considering 16 weeks as the lower cut-off of preterm birth? Further, one of the inclusion criteria is *“presentation to antenatal care prior to 20 weeks of gestation if HIV-uninfected or 24 weeks if HIV-infected”.* Nearly 50% of the women first present to antenatal care after 16 weeks in this population and 25% above 18.3w (median EGA at enrolment is 16.1 (IQR: 13.3–18.3)w). This means there would be some pregnant women in the population who delivered preterm (between 16 & 24w) who couldn’t be a part of the cohort because of the delay in seeking antenatal care. Would that create a bias? If so, how much would this influence the study’s estimate of the preterm birth rate in the population? Reporting preterm birth rate among the participants enrolled less than 16 weeks of gestation will add further information in this aspect. 

 

 The manuscript can be accepted for indexing after the resolution of the comments made above.

I confirm that I have read this submission and believe that I have an appropriate level of expertise to confirm that it is of an acceptable scientific standard, however I have significant reservations, as outlined above.

# Response

**Published:** 2019-11-14

**Authors:** Joan Price

We chose to include preterm stillbirths in our definition of preterm birth and demonstrate the overlapping adverse outcomes of preterm birth, stillbirth, and small for gestational age in Figure 2. We acknowledge significant limitations in differentiating intrapartum stillbirth from preterm birth with immediate neonatal death in our setting. We believe that to exclude stillbirths that occur in the process of parturition from spontaneous preterm birth would falsely lower the rate of spontaneous preterm delivery. As we note in the discussion, the categorization of antepartum vs. intrapartum stillbirth is also imperfect. Of 181 preterm deliveries in our cohort, 48 (27%) were stillborn. Excluding stillbirths from our preterm delivery definition reduced preterm delivery from 15% to 11% and spontaneous preterm delivery from 10% to 8%. Despite this overall reduction, excluding stillbirths from our regression models did not significantly alter our estimates of risk, which supports evidence that risk factors for live and stillborn preterm births demonstrate substantial overlap (Kramer 2012). Finally, our cohort was designed to evaluate the risk factors associated with adverse birth outcomes, and not necessarily to estimate population-level rates of these outcomes. We note in the discussion that our study population likely over-represents high-risk women and therefore overestimates the true population incidence of adverse birth outcomes. 

Both lower and upper EGA boundaries for defining preterm birth vary widely in research and national statistics worldwide. Participants at the International Conference on Prematurity and Stillbirth of the Global Alliance to Prevent Prematurity and Stillbirth (GAPPS) have argued for including births occurring 16 weeks onward in the definition of preterm birth, citing studies that show similar etiological risk factors for births occurring as early as 16 weeks and those that occur later in the 2 ^nd^ and 3 ^rd^ trimester (Kramer 2012; Villar 2012). We acknowledge that the risk of preterm birth in any cohort increases with earlier gestational age at presentation for precisely the reason explained by the reviewer. There is a strong relationship between EGA at presentation and EGA at delivery, which therefore produces this effect regardless of relatively arbitrary EGA boundaries used to define outcomes. Indeed, the preterm birth rate among participants who enrolled <16 weeks was 18% in our cohort compared to 12% among those enrolled ≥16 weeks. Because of this, we include EGA at enrollment in all multivariable analyses. We also repeated analyses of preterm delivery outcomes restricting our sample to those participants who presented <20 weeks and again to those who presented <16 weeks to evaluate the potential for bias. These restricted analyses had no effect on our results (with the exception of short cervix as an exposure since it was only performed beyond 16 weeks).

# Peer review: Reviewer response for version 1

**DOI:** 10.21956/gatesopenres.14168.r28081

**Published:** 2019-11-04

**Authors:** Ge Zhang

**Affiliations:** 1 Division of Human Genetics, Cincinnati Children’s Hospital Medical Center, University of Cincinnati College of Medicine, Cincinnati, OH, USA

This is an important study and the data presented adds new knowledge about the adverse birth outcomes and their risk factors in low-income countries in sub-Saharan Africa. The advantages of the study include:

1. Well-designed and well-conducted cohort study;
2. Reliable gestational age dating and clinical phenotyping;
3. Well-defined primary exposures and birth outcomes;
4. Distinction between spontaneous and provider-initiated PTB;
5. Comprehensive and reliable data analysis;
6. Results were well-organized clearly presented;
7. Limitations were well noted and discussed.

Minor comments:

1. The reviewer recognized that the authors tried to avoid using p-values in this report according to recent guidelines and recommendations. However, sometimes it could be helpful to the readers to comprehend the “substantiality” of the statistical evidence. The reviewer therefore would suggest including p-values in Figure 3, Table 2, etc. (as Table 1).
2. EGA at enrollment (<14) was shown to be associated with PTB. The authors might compare the baseline characteristics between the samples enrolled before and after 14wks to identify the possible reasons underlying this association (similar to the comparisons made in Table 1). In addition, as the gestational age was calculated differently in the samples enrolled before and after 14wks, the authors might also compare outcomes (e.g. gestation duration) between these two groups to examine whether the two methods could potentially cause systematical difference in the estimation of gestational age.
3. The authors presented the co-occurrence among PTB, SGA and SB using a Venn diagram (Figure 2). It is also interesting to learn whether the frequencies of the co-occurrence of these outcomes were higher than expected especially between the very PTB and very SGA group.
4. The authors dichotomized continuous exposures (e.g. maternal age, BMI) as well as the outcomes (e.g. PTB, SGA) in the association analysis (Table 3). It would be more informative if the authors could also show the results based on association tests of continuous variables.
5. Maternal height has been shown to be associated with gestational duration and birth weight (gestational age adjusted) in previous studies mainly in high-income countries. It is not known whether maternal height is associated with gestational duration (and PTB) in this study or not.
6. In the discussion section, the authors should compare the risk factors (e.g. their occurrence, frequencies and estimated effect sizes) and the frequencies of adverse birth outcomes reported in this study with those reported in high-income countries.

I confirm that I have read this submission and believe that I have an appropriate level of expertise to confirm that it is of an acceptable scientific standard.

# Response

**Published:** 2020-02-07

**Authors:** Joan Price

The authors thank the reviewer for the thoughtful and constructive comments. Please find a point-by-point response to each comment here. 

1. *We have elected not to include *p *values in our tables beyond Table 1 because the we think point estimates and their 95% confidence intervals are the best estimates of association. (see Harrington et al NEJM 2019 https://www.nejm.org/doi/full/10.1056/NEJMe1906559).  *
2. *Thank you for this suggestion. In response to the other reviewer’s comment regarding the increased risk of PTB with EGA at enrollment, we have repeated analyses of preterm delivery outcomes restricting our sample to those participants who presented <20 weeks and again those who presented <16 weeks to evaluate the potential for bias and our results proved stable. We also include EGA at enrollment in all multivariable analyses. We think that additional reporting of which baseline characteristics differ by EGA at enrollment and investigation of the effect of ultrasound algorithms used for EGA calculation is beyond the scope (and page limit) of this analysis but may be explored in future analyses. *
3. *Thank you for this suggestion. We have added a second Euler diagram to Figure 2 to illustrate the co-occurrence of very PTB, very SGA, and stillbirth.*
4. *We have added linear regression of continuous exposures and continuous outcomes (Table 6). *
5. *We have noted in the results section that maternal height was not associated with either PTB or SGA. It was similarly not associated with gestational duration.*
6. *We have added additional global context to our estimates of the frequencies of adverse birth outcomes in the discussion section. However, we acknowledge significant limitations to direct comparisons of risk estimates due to inconsistent definitions. Furthermore, as there is still no undisputed global standard by which to identify small for gestational age neonates, we have not directly compared the incidence of SGA in our cohort to estimates from high-income countries. *
